# Targeting and monitoring mean arterial pressure in critical illness: A mixed-methods service evaluation

**DOI:** 10.1177/17511437261415835

**Published:** 2026-02-14

**Authors:** Isla MacKay, Ian Piper, Annemarie B. Docherty

**Affiliations:** 1 Edinburgh Medical School, University of Edinburgh, U; 2 Centre for Medical Informatics, Usher Institute, University of Edinburgh, UK

**Keywords:** mean arterial pressure, blood pressure, hypotension, haemodynamic stability

## Abstract

**Background:**

In sepsis and cardiac arrest, arterial hypotension is associated with poorer outcomes, including renal injury and mortality. Guidelines recommend a mean arterial pressure (MAP) target of ⩾65 mmHg, but supporting evidence is limited. We undertook a service evaluation which aimed to: (1) assess clinical opinion regarding the optimal MAP target in intensive care (ICU); and (2) evaluate MAP target adherence at the Royal Infirmary Edinburgh ICU, quantifying levels of hypotension.

**Methods:**

We utilised a concurrent triangulation mixed-methods approach, integrating semi-structured consultant interviews and quantitative analysis of patient-level blood pressure data. Blood pressure data were collected at 1-min intervals for the first 72 h of arterial monitoring. We defined hypotensive insults by five sequential minutes below MAP target.

**Results:**

We interviewed 18 consultants. Twelve (67%) reported a standard target of 65 mmHg. The importance of evidence-based, individualised, and flexible targets was emphasised. We included 208,570 min of monitoring time across 66 patients. At admission, 53 (80%) patients received a target of 65 mmHg. Mean (SD) MAP was lower in patients on vasopressors than those not on vasopressors (77.6 (14.2) vs 86.9 (17.3) mmHg, *p* = 0.0001). Hypotension affected 55 (83%) patients and accounted for >10% of monitoring time in thirteen (20%). Median pressure-time index (PTI) was 3.4 mmHg * h; 24 (36%) patients had a PTI >10 mmHg * h.

**Conclusions:**

The optimal MAP target varied by patient, yet target personalisation remained limited in practice. Target adherence varied, with observed MAP both exceeding and undershooting set targets. Future research will explore the feasibility and implications of achieving tighter blood pressure control.

## Introduction

Hypotension has various definitions, but in the Intensive Care Unit (ICU), it is most commonly defined as a mean arterial pressure (MAP) below 65 mmHg.^1^ As a cardinal feature of shock, hypotension has repeatedly been associated with worse clinical outcomes, including renal injury and mortality.^2,3^

Treatment of hypotension currently involves increasing preload through fluid resuscitation, myocardial contractility through inotropes, and/or systemic vascular resistance through vasopressors.^4^ MAP is used to guide delivery of these agents, with the Surviving Sepsis Campaign and European Critical Care Society guidelines recommending initial resuscitation to a MAP of at least 65 mmHg in septic or cardiogenic shock.^5,6^ However, with little foundation in clinical trial evidence, this recommendation has faced dispute.^7^

Vasopressor use also carries risk, most notably of myocardial and ischaemic injury.^8,9^ Balancing these risks with those of hypotension remains a challenge, particularly given the diversity of ICU patients. There is a growing suggestion that the optimal MAP target varies on an individual basis, for example, based on cardiovascular history or patient age.^10^

Patients with cardiovascular disease have a vulnerable myocardium that is at risk of oxygen supply-demand imbalance during critical illness. There is clinical rationale that these patients may require higher targets to maintain coronary perfusion.^11^ Alternatively, increased exposure to beta-agonist drugs may increase left ventricular work and risk of arrhythmia.^8^ Similarly, among patients with chronic hypertension, there is evidence to suggest that higher pressures improve renal function and may be required for neuroprotection.^12,13^

There is some evidence favouring lower MAP targets in older patients. Most notably, a large randomised controlled trial suggested that a permissive hypotension strategy in over-65s with septic shock is likely safe and perhaps advantageous.^14^ This strategy reduces exposure to exogenous vasopressors and could avoid overwhelming older patients’ more limited physiological reserve.

Ultimately, the optimal MAP target remains unclear. We undertook a service evaluation which aimed to: (1) assess clinical opinion regarding the optimal MAP target in critical illness; and (2) evaluate MAP target adherence at the Royal Infirmary Edinburgh ICU, quantifying levels of hypotension among patients with non-neurological primary diagnoses in this unit.

## Methods

This service evaluation utilised a concurrent triangulation mixed-methods approach, integrating qualitative analysis of semi-structured interviews with critical care consultants and quantitative analysis of patient-level arterial blood pressure data. The study was part of a quality improvement programme of work, with the interviews being part of the process to develop ideas for change. Caldicott approval was given by the NHS Lothian Critical Care Quality Improvement Team.

### Consultant interviews

#### Participants and setting

Semi-structured interviews (I. MacKay) were conducted with consultants in critical care at the Royal Infirmary, Edinburgh. Whilst the interviewer had no formal interview experience, they consulted social scientists within the group to design interview structure and discuss techniques on how to draw out key themes. Convenience sampling was employed, wherein participants were approached whilst working in the ICU. Recruitment continued until saturation of themes was reached.

#### Data collection

Interviews were conducted on-site and were of 10 min duration. Since ethical approval for audio recording was not sought, interviews were manually annotated by the interviewer.

The semi-structured design provided a level of consistency across interviews whilst allowing for flexible and detailed discussion. A three-part interview guide was developed following a literature review of MAP trials. Questions focused on establishing: (1) consultants’ standard MAP targets for non-neurological ICU patients; (2) clinical factors influencing their targets; and (3) any challenges in setting MAP goals.

#### Analysis

Interview texts were anonymised and underwent an inductive thematic analysis, using NVivo 14 and an analytical scaffold described by Nowell et al.^15^ This involved manually coding the data and grouping codes into common themes. The interviewer also handled analysis responsibilities.

### Quantitative analysis of mean arterial pressure

We conducted a single-centre service evaluation in the Royal Infirmary Edinburgh ICU between January 24, 2024, and March 6, 2024, for adult (⩾18 years) patients receiving level 3 ICU care. We excluded patients with a neurological diagnosis, those receiving palliative care, and those without arterial pressure monitoring.

We collected physiological data at 1-min intervals (heart rate, systolic blood pressure, diastolic blood pressure, MAP, oxygen saturation) for the first 72 h of arterial line monitoring, where length of stay allowed, daily vasopressor doses and exposure hours (expressed as a noradrenaline equivalent, calculated through validated formulae^16^), and demographic data (age, sex, comorbidities, and admission diagnosis). Individual MAP targets were collected from medical notes and corroborated through discussions with nurses. If admitting MAP target was altered during the monitoring period, this was recorded. No formal power calculations were performed. Sample size was limited by patient turnover and a short recruitment window. Physiological data were extracted from the Mindray central monitoring system.^17^ All data were pseudo-anonymised, and stored and analysed on a password-protected NHS computer, to which only the researchers had access.

In accordance with the Edinburgh University Secondary Insult Grading (EUSIG) scheme, we defined hypotensive insults by five sequential minutes below MAP target.^18^ To mitigate artefacts, such as detached probes or line flushing, we discarded episodes shorter than 5 min. The cumulative time below threshold that did not reach our 5-min rule was used as a surrogate measure of length of invalid data.

Insults were described in terms of number, absolute duration, as a percentage of total valid monitoring time, and through a pressure-time index (PTI). The PTI is the cumulative area under the threshold, normalised per hour of insult.^18^ It integrates both the depth and duration of hypotensive insults, with deeper hypotension producing a greater PTI when comparing insults of equal duration but varying severity.

We defined hypertensive insults by five sequential minutes above a MAP of 110 mmHg, an accepted threshold for hypertension.^19^

All statistical analyses were performed using Rstudio (R Version 4.3.2) and a 5% significance threshold. Categorical data were expressed as a total (percentage) and compared using Pearson’s Chi-squared test or Fisher’s exact test. Non-parametric continuous variables were expressed as a median (quartile one, quartile three) and were compared using the Mann-Whitney *U* test or Kruskal Wallis’ test. To identify clinical factors associated with hypotension or vasopressor requirement, multivariable logistic regression analysis was employed. Age, sex, admitting diagnosis, chronic hypertension, ischaemic heart disease, and liver cirrhosis were entered as covariates.

## Results

### Consultant interviews

Twenty-five (63%) of a potential 40 critical care consultants were contacted for interview. All but five were invited face-to-face, with the rest contacted via email (snowball recruitment). Eighteen consultants participated in interviews.

Three key themes emerged from thematic analysis of interview data: evidence-based targets; individualised targets; and flexible targets. Key themes represent ideas that were largely emphasised and echoed by interviewees.

#### Theme 1: Evidence-based targets

Several participants emphasised the importance of practicing evidence-based medicine. Twelve (67%) said they use a MAP target of 65 mmHg as standard, as guidelines advise (Table 1, Statement A).

**Table 1.** Extracts from consultant interviews.

Reference	Clinician comments
Theme 1	Evidence-based targets
A	“*It is important to use trial-based evidence . . . use MAP 65 [mmHg] as a general guideline*.”
B	“*If over 65 and in septic shock, I would set my target at 60 [mmHg], as the 65-Trial suggests*.”
C	“*We don’t have enough recent or strong evidence to base MAP targets on . . . lots of guidelines stem from old physiology textbooks or research of heterogeneous populations.*”
Theme 2	Individualised targets
D	“*My target depends on the patient and a combination of clinical, surgical, and laboratory parameters.*”
E	“*I would consider raising my target to around 70 [mmHg] in chronic hypertension, although there is limited evidence to support this.*”
F	“*In aortic stenosis, cardiac output is fixed so the risks of hypoperfusion are great. It is important to aim higher to avoid a catastrophic spiral of hypoperfusion*.”
G	“*In Type B aortic dissection, a permissive hypotension strategy helps to avoid propagation of the dissection.*”
H	“*In aortic regurgitation, higher MAPs can increase regurgitant volume. I advise setting a lower target of around 55mmHg and monitoring response through an echo[cardiogram].*”
I	“*If young and fit, I would accept a lower target of 55–60 [mmHg]. If older, I would avoid challenging the patient by raising my target . . . older patients are more likely to be chronically hypertensive.*”
J	“*I may trial a higher MAP in acute kidney injury if urine output is low, but this likely won’t work. . . the brain and the heart are the main organs to titrate MAP against.*”
K	“*In cardiogenic shock, avoid putting unnecessary pressure on a vulnerable heart by accepting a lower MAP.*”
L	“*In hypovolaemic shock, be careful not to push high-dose vasopressors without considering fluid status . . . and whether the patient actually requires a MAP of 65*.”
M	“*Identify underlying aetiology and tailor blood pressure targets to it.*”
Theme 3	Flexible targets
N	“*There is too much focus on a single number that doesn’t tell you much about how much oxygen an organ system is actually receiving.*”
O	“*Trial different MAPs and [vasopressor] doses and see how the patient tolerates them.*”
P	“*MAP is only a surrogate marker of perfusion . . . If cerebrating and peeing but MAP is low, so what? Why keep chasing a single number?*”

MAP: mean arterial pressure.

Some participants referenced landmark MAP trials: the 65-Trial and SEPSISPAM.^12,14^ They said that this evidence could be useful when managing elderly patients or patients with chronic hypertension. Specific blood pressure protocols for traumatic brain injury, aortic dissection, and active haemorrhage were also referenced^20–22^ (Table 1, Statement B).

However, the decision-making process was not strictly dictated by clinical trial evidence or guidelines. Whilst many acknowledged a lack of evidence for targets other than 65 mmHg, six (33%) interviewees said they used alternative thresholds as their standard. Some questioned the quality of clinical trial evidence and highlighted the need to balance clinical judgement with available evidence as crucial (Table 1, Statement C).

#### Theme 2: Individualised targets

There was a general consensus that a single target was not universally suitable for all patients. Participants described several factors that informed their decision-making, including cardiovascular history, patient age, renal function, type of shock, and underlying aetiology (Table 1, Statement D).

##### Cardiovascular history

Participants noted that cardiovascular needs were unique to each patient. Notably, 16 (89%) participants agreed that patients with chronic hypertension may require higher targets since pre-morbid blood pressure was raised. The severity and control of hypertension, as well as the presence of active ischaemic heart disease, were also highlighted as important considerations (Table 1, Statement E). Other relevant cardiovascular conditions included aortic stenosis, aortic dissection, aortic regurgitation, and thoraco-abdominal aortic aneurysm (Table 1, Statement F–H).

##### Patient age

Questions around patient age divided clinical opinion, with age holding no significance for half of the participants. Some advocated for a permissive hypotension strategy in over-65s with septic shock, as the 65-Trial suggested.^14^ Others suggested that higher targets were more appropriate in older patients, who were more likely to have relevant cardiovascular history (Table 1, Statement I).

##### Renal function

Although not a primary consideration, 13 (56%) participants said they would consider renal function when targeting MAP. Many highlighted urine output as a key perfusion marker. Some noted that in pre-renal acute kidney injury, trialling higher MAPs may improve urine output (Table 1, Statement J).

##### Type of shock

Participants acknowledged that different types of shock presented different challenges. In cardiogenic shock, focus lay on minimising exposure to beta-agonist drugs that increase myocardial work. This could involve accepting lower targets (Table 1, Statement K). Equally, participants suggested that lower targets may be acceptable in septic shock if the patient was otherwise stable. There was recognition of the role of fluid resuscitation in septic shock, and of a permissive hypotension strategy in controlling bleeding in active haemorrhage (Table 1, Statement L).

##### Underlying aetiology

All participants described scenarios where they would tailor MAP to admitting diagnosis. They noted that neurological conditions, including stroke, traumatic brain injury, and subarachnoid haemorrhage, may necessitate higher pressures to maintain cerebral perfusion. Various vascular pathologies were also of relevance, including aortic dissection, aortic stenosis, thoraco-abdominal aortic aneurysm, and post-vascular surgery situations. Some described targeting lower pressures in liver cirrhosis to account for a chronically lower blood pressure (Table 1, Statement M).

#### Theme 3: Flexible targets

Several participants challenged the singular focus on MAP and emphasised the importance of assessing broader clinical, laboratory, and surgical markers of perfusion. Discerning between blood flow and perfusion pressure, they stressed that MAP is only a surrogate marker of perfusion (Table 1, Statement N).

Participants described using a trial-and-error approach, where targets are adjusted based on patient response. Many questioned the necessity of strictly adhering to a specific target if other signs of adequate perfusion were present. Other perfusion markers identified included: urine output, mental status, lactate levels, capillary refill time, and cardiac output (Table 1, Statements O, P).

## Arterial monitoring

Of the 102 patients assessed for eligibility, 66 were included, with a total of 208,570 min of monitoring time collected (Table 2). All patients had a MAP target recorded in their medical notes. The bedside nurse was aware of this target in 62 (94%) cases. A bedside poster documented this target in 12 (18%) cases. Fifty-three (80%) patients received an admission MAP target of 65 mmHg, range 60–100 mmHg. Admission targets were adjusted for 10 (15%) patients during the 72-h monitoring period. Targets were similar across all patient subgroups, irrespective of comorbidity, admitting diagnosis, or age (Table 3).

**Table 2.** Baseline characteristics stratified by vasopressor use.

Characteristic	Received vasopressors *n* = 42	Did not receive vasopressors *n* = 24	Total *n* = 66
Age; y	56 (42, 68)	53 (38, 60)	54 (36, 68)
Sex; male	27 (64%)	16 (67%)	43 (65%)
Comorbidities			
Chronic hypertension	13 (31%)	11 (46%)	10 (22%)
Ischaemic heart disease	7 (17%)	1 (4%)	36 (55%)
Heart failure	3 (7%)	1 (4%)	4 (6%)
Liver cirrhosis	9 (21%)	3 (13%)	12 (18%)
Chronic kidney disease	6 (14%)	2 (8%)	8 (12%)
Primary diagnosis at ICU admission			
Gastrointestinal	15 (36%)	6 (25%)	21 (32%)
Cardiovascular	13 (31%)	0 (0%)	13 (20%)
Respiratory	3 (7%)	9 (38%)	12 (18%)
Metabolic	7 (17%)	5 (21%)	12 (18%)
Sepsis	3 (7%)	0 (0%)	3 (5%)
Other	1 (2%)	4 (17%)	5 (8%)
Post-operative	18 (43%)	9 (38%)	27 (41%)
Initial MAP target; mmHg			
60	2 (5%)	2 (8%)	4 (6%)
65	34 (81%)	19 (79%)	53 (80%)
70	2 (5%)	2 (8%)	4 (6%)
75 or above	4 (10%)	1 (4%)	5 (8%)
Noradrenaline-equivalent vasopressor dose; mg		–	–
Total dose	18.8 (6.2, 51.0)	–	–
Day 1 dose	11.0 (3.6, 23.4)	–	–
Day 2 dose	5.1 (1.6, 15.3)	–	–
Day 3 dose	0.7 (0.0, 10.6)	–	–

HTN: hypertension; IHD: ischaemic heart disease.

Values are *n*(%) or median (*Q*1, *Q*3).

**Table 3.** Admission MAP targets stratified by baseline characteristics.

Characteristic	*n*	Admission MAP target; mmHg			*p*-Value
		<65	65	>65	
Age; y					
<65	46	3 (7%)	37 (80%)	6 (13%)	0.7
⩾65	20	1 (5%)	16 (80%)	3 (15%)	–
Sex					
Male	43	3 (7%)	33 (77%)	7 (16%)	0.5
Female	23	1 (4%)	20 (87%)	2 (9%)	–
Comorbidities					
Chronic hypertension	24	3 (13%)	17 (71%)	4 (17%)	0.2
Ischaemic heart disease	8	0 (0%)	6 (75%)	2 (25%)	0.3
Liver cirrhosis	13	1 (8%)	11 (85%)	1 (8%)	0.9
Primary diagnosis at ICU admission					
Gastrointestinal	22	1 (5%)	18 (82%)	3 (14%)	0.7
Cardiovascular	13	0 (0%)	11 (85%)	2 (25%)	0.5
Respiratory	12	1 (8%)	10 (83%)	1 (8%)	1.0
Metabolic	13	1 (8%)	11 (85%)	1 (8%)	0.9
Sepsis	3	1 (33%)	2 (66%)	0 (0%)	0.2
Post-operative					
Yes	27	3 (11%)	19 (73%)	5 (19%)	0.1
No	39	1 (3%)	34 (87%)	4 (10%)	–

MAP: mean arterial pressure; ICU: intensive care unit.

Values are *n*(%).

Forty-two (64%) patients received vasopressors, most commonly noradrenaline only (Table 4). There were no differences in age, sex, comorbidity, or diagnosis between vasopressor and non-vasopressor groups (Table 2). The median duration of exposure to vasopressors was 47 h (IQR 27, 64). Dosing was highest on day 1 (noradrenaline equivalent dose, 18.8 mg (IQR 6.2, 51.0)) and gradually weaned across the 3 days (Table 4).

**Table 4.** Vasopressor use during the first 72 h of ICU admission (*n* = 42).

Variable	Vasopressor group *n* = 42
Noradrenaline-equivalent vasopressor doses; mg	
Total dose	18.8 (6.2, 51.0)
Day 1 dose	11.0 (3.6, 23.4)
Day 2 dose	5.1 (1.6, 15.3)
Day 3 dose	0.7 (0.0, 10.6)
Vasopressor timings	
Duration of exposure; h	47 (27, 64)
Time to first dose; min	58 (0, 314)
Vasopressor type	
Noradrenaline only	26 (62%)
Metaraminol only	2 (5%)
Combination	14 (33%)
Inotrope type	
Isoprenaline only	2 (5%)
Milrinone only	1 (2%)
Other or combination	2 (5%)

Values are median (*Q*1, *Q*3) or *n*(%). Noradrenaline equivalent doses were calculated as follows (Ref. 16): adrenaline µg/kg/min (×1), vasopressin U/min (×2.5), and metaraminol µg/kg/min (×0.8).

**Figure 1.**
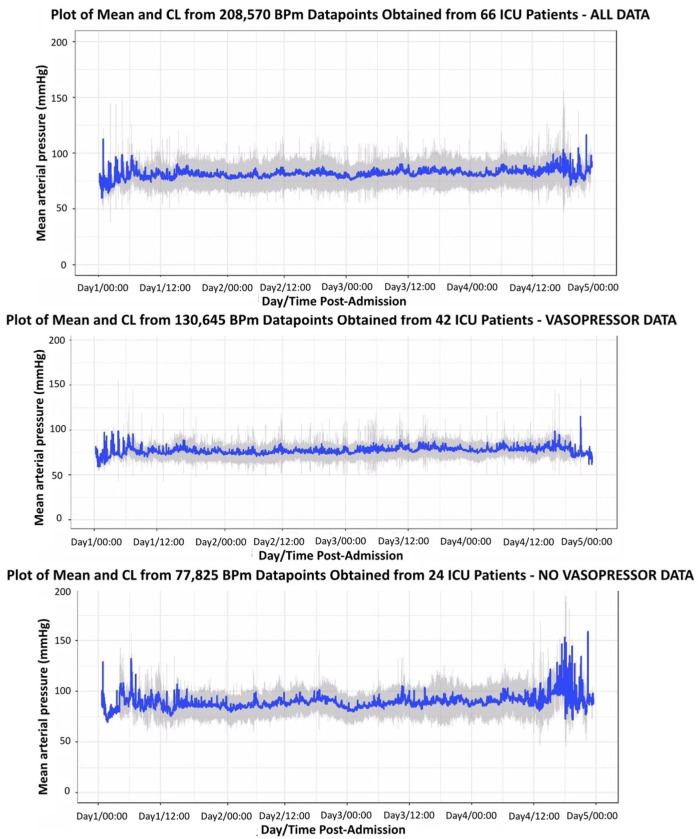
Plots of mean arterial pressure across the first 72 h of admission, stratified by vasopressor status. The 95% confidence interval (grey) around the mean MAP (blue) for the first 72 h of admission is illustrated. Data were filtered to exclude MAP values >250 and <30 mmHg and aligned to real-time minutes post-admission. CL: confidence limits; ICU: intensive care unit; BPm: mean arterial pressure.

Across all patients, the mean (SD) MAP was 81.5 (16.3) mmHg. Mean (SD) observed MAP was lower in patients who received vasopressors compared to those who did not (77.6 (14.2) vs 86.9 (17.3) mmHg, *p* = 0.0001; Figure 1). Four (10%) patients in the vasopressor group received an admission MAP target above 75 mmHg.

**Figure 2.**
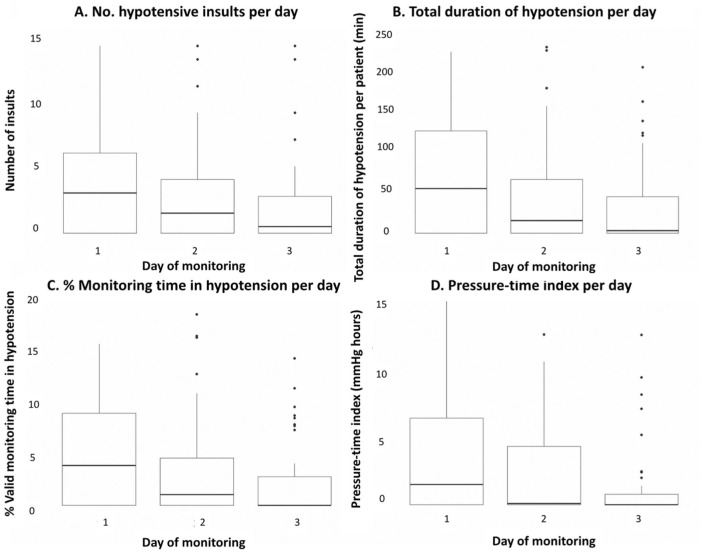
Distribution of hypotension by day of admission. The burden of hypotension is expressed in terms of (a) number of hypotensive insults, (b) cumulative duration of insults, (c) as a proportion of valid monitoring time, and (d) through a pressure-time index (PTI).

Fifty-five (83%) patients experienced at least one hypotensive insult, with the highest incidence of insults occurring on day 1 of admission (Figure 2). On average, hypotensive insults accounted for 3.3% (0.5%, 7.3%) of total valid monitoring time. Thirteen (20%) patients spent greater than 10% of their monitoring time in hypotension. Among these 13, the median PTI on days 1–3 was 7.9 (6.3, 20.0), 9.1 (1.6, 14.4), and 4.6 (0.0, 7.5), respectively. The median 72-h PTI for all patients was 3.4 (0.4, 14.0) mmHg * h. Twenty-four (36%) patients had a PTI over 10 mmHg * h.

Forty (61%) patients experienced at least one hypertensive insult. Hypertensive episodes accounted for 0.3% (0.0%, 2.4%) of total valid monitoring time and the median PTI for hypertension was 0.2 (0.0, 21.5) mmHg * h.

**Figure 3.**
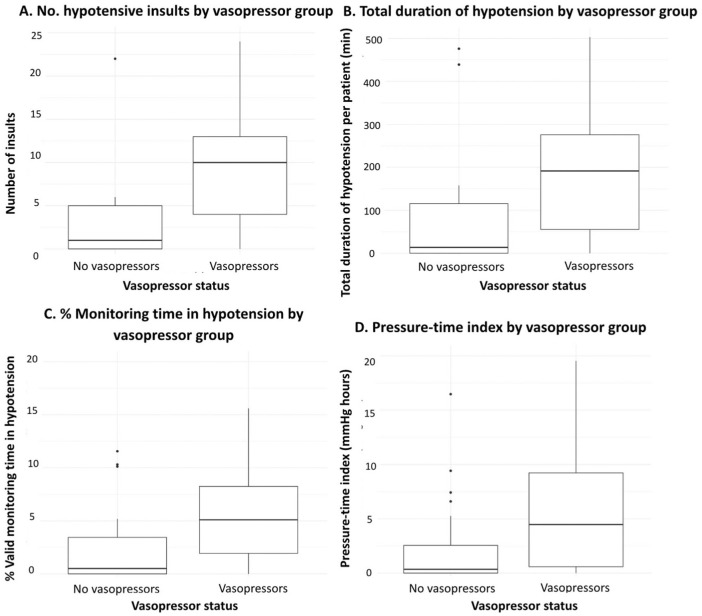
Hypotension exposure stratified by vasopressor status. The burden of hypotension is expressed in terms of (a) number of insults, (b) cumulative duration of insults, (c) as a proportion of valid monitoring time, and (d) through a pressure-time index (PTI).

**Figure 4.**
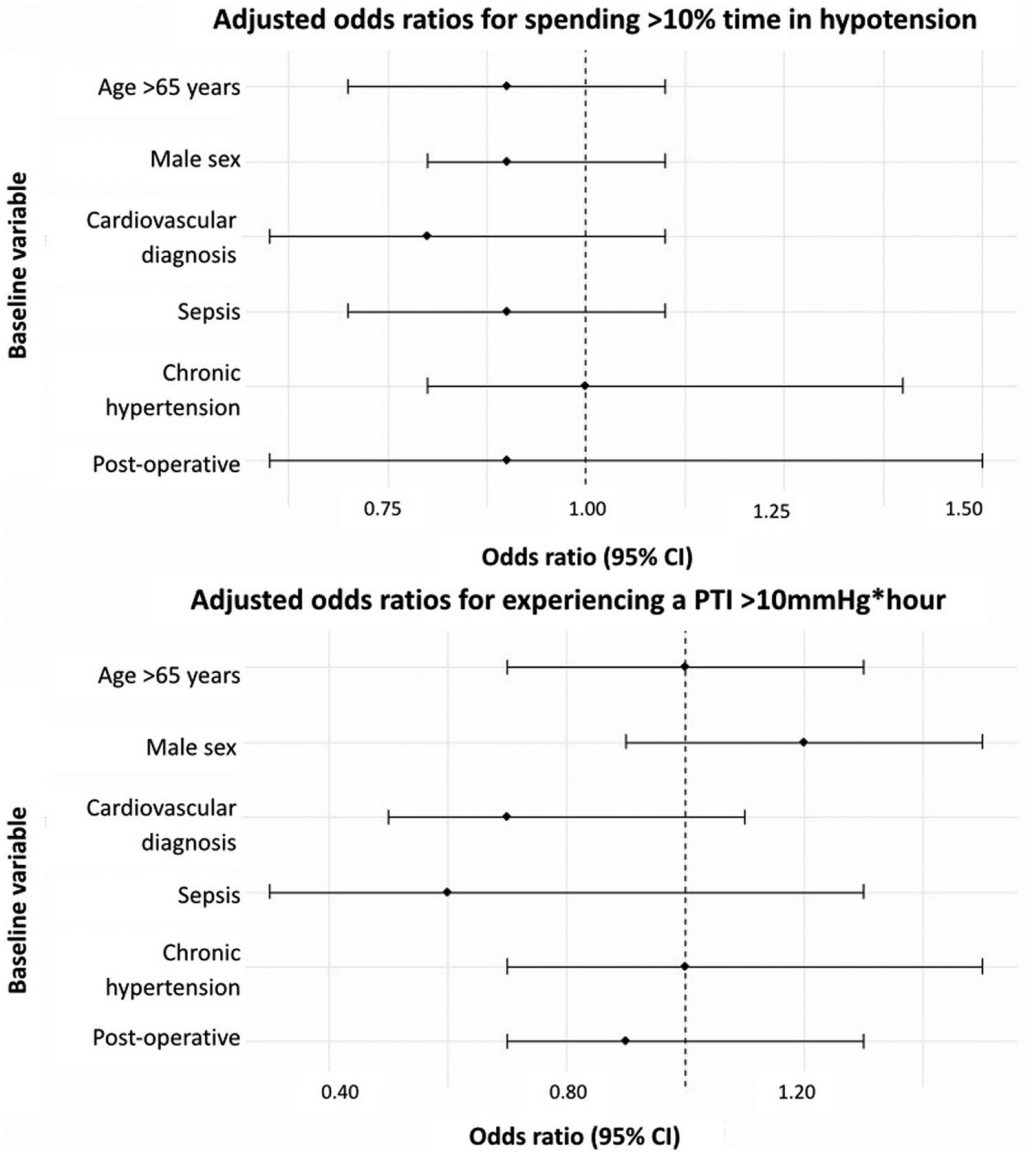
Forest plots of the clinical factors associated with hypotension. Multivariable logistic regression analysis employed age, sex, admitting diagnosis, surgical status, and comorbidities as covariates. PTI: pressure-time index.

Patients on vasopressors experienced more hypotensive insults per patient (10 vs 2, *p* = 0.001), a longer absolute duration of hypotension per patient (192 vs 41 min, *p* = 0.01), a greater proportion of valid monitoring time spent below target (5.1 vs 1.0%, *p* = 0.01), and higher PTI values (9.0 vs 1.1 mmHg * h, *p* = 0.01; Table 5, Figure 3). Patients on vasopressors experienced less hypertension. No other clinical factors were associated with hypotension (Figure 4).

**Table 5.** Burden of hypotension stratified by vasopressor status.

Hypotension measure^a^	Vasopressor group, *n* = 42	No vasopressor group, *n* = 24	Difference in medians (95% CI)	*p*-Value
Number of insults	10 (4, 13)	2 (0, 5)	8 (6–13)	0.001
Duration; min	192 (55, 276)	41 (0, 45)	151 (89–260)	0.01
Proportion of time; %^b^	5.1 (1.9, 8.2)	1.0 (0.0, 4.1)	4.1 (2.7–7.2)	0.01
PTI; mmHg * h^c^	9.0 (1.2, 18.5)	1.1 (0.0, 7.3)	7.9 (2.7–15.6)	0.01

PTI: pressure-time index; CI: confidence interval.

Values are median (*Q*1, *Q*3) or difference in medians (95% confidence interval).

a Values represent the median burden of hypotension per patient in vasopressor and non-vasopressor groups.

b Cumulative duration of hypotensive insults expressed as a proportion of total valid monitoring time.

c The pressure-time index (PTI) integrates the depth and duration of hypotensive insults. It was calculated according to the method described by Jones et al.^18^

## Discussion

There is limited evidence behind current MAP guidelines and a growing suggestion that the optimal target varies from patient-to-patient.^7^ Our service evaluation contributes to the ongoing discourse on MAP management by describing clinical opinion around the optimal target and auditing compliance with these targets. It is novel in that it uses a mixed-methods approach to examine whether clinical opinions were effectively translated into practice.

### Evidence-based targets

Interviews suggested variable guideline compliance.^5,6^ Whilst participants acknowledged a lack of evidence for targets other than 65 mmHg, a third used alternative thresholds as their standard. Although few cited landmark trials (e.g. 65-Trial, SEPSISPAM), those who did highlighted their relevance to older or chronically hypertensive patients.^12,14^ Others questioned the quality of trial evidence, highlighting that the current evidence-base is derived from trials of heterogeneous populations that may lack the power to detect clinically significant subgroup effects.

### Individualised targets

Consultants cautioned against a one-size-fits-all approach, questioning the rationale behind a singular MAP goal for diverse patients. Cardiovascular history, age, renal function, type of shock, and admitting diagnosis emerged as important considerations when managing MAP.

There was particular emphasis on tailoring targets to baseline blood pressure: lowering targets in liver cirrhosis and raising targets in chronic hypertension. A right-shift of cerebral autoregulation has been observed in chronic hypertension, suggesting that higher pressures are required for neuroprotection.^13^ Similar autoregulatory adaptations can occur in other organs.^23–25^ However, there is limited evidence supporting this strategy. Whilst subgroup analysis from the SEPSISPAM trial found that lower MAPs increased need for renal replacement therapy in patients with chronic hypertension (42.2% vs 31.7%, *p* = 0.04), this finding has not been replicated since.^12,14,26–31^

Patient age held variable significance for interviewees. Some recommended a permissive hypotension strategy in over-65s with septic shock, as the 65-Trial suggests.^14^ This strategy reduces vasopressor use and acknowledges that excessively aggressive attempts to normalise blood pressure may strain an older heart. Alternatively, some recommended higher targets in older age, where co-morbid hypertension is more likely.

Admitting diagnosis emerged as a primary factor influencing MAP goals. As per protocol, various aortic pathologies, including dissections, aneurysm, stenosis, and regurgitation, were described as requiring bespoke targets.^20–22^

### Flexible targets

Flexibility in approach, particularly when patients are struggling to achieve a particular MAP, was highlighted as crucial. Rather than strictly adhering to pre-defined thresholds, consultants encouraged assessment of broader markers of perfusion: cognitive function, urine output, and mental status. Noting that MAP is only a surrogate marker of perfusion, they questioned the necessity of chasing a specific MAP where other perfusion markers were present.

Despite interview suggestions, we observed limited personalisation of targets in practice, with similar targets across all patient subgroups. Whilst a small proportion of interviewees did not care for our patient cohort, this alone is unlikely to explain the discrepancy between interview and clinical data. Obstacles to target personalisation, such as insufficient information regarding baseline blood pressure, require further investigation.

Although not to the extent that interviews might suggest, some target individualisation was observed, with 15% of patients receiving admitting targets other than 65 mmHg. These ranged from 60 to 100 mmHg. There was also evidence of flexibility in approach, wherein 10 patients’ targets were adjusted based on renal or cognitive response.

Observed MAP was typically higher than targeted in the vasopressor group, raising concerns around target adherence and the potential overuse of exogenous vasopressors. Conversely, hypotensive insults were common, affecting 84% of our cohort. Despite our conservative analytical approach, this rate is higher than reported incidences in previous ICU studies, which range from 47% to 72%.^32–35^ Hypotension accounted for approximately 3% of total patient monitoring time, or 2.5 h of hypotension for a 72-h stay. Median PTI was 3.4 mmHg * h, meaning that, on average, MAP was 3.4 mmHg below target per hour of hypotension. Given the mild nature of this hypotension, and evidence supporting a permissive hypotension strategy in certain patients, duration figures may cause undue concern.^14^ However, it is worth noting that PTI varied substantially between patients, with 36% experiencing a PTI greater than 10 mmHg * h.

An international survey of ICU personnel suggested that ICU hypotension is under-diagnosed and largely preventable.^4^ Timely recognition of hypotension was identified as a particular issue. Future work into the development of improved secondary insult visualisation approaches and predictive algorithms could facilitate pre-emptive intervention.^36,37^ However, it is important to remember that the relationship between hypotension and ICU outcomes remains complex. Hypotension can be a marker of underlying illness severity and correcting it may not always improve outcomes.^38^

This service evaluation has limitations. A key weakness is its small and single-centred nature, with patient turnover and a short recruitment window having limited size. Second, independent recording and analysis of interviews could have facilitated a more nuanced analysis and mitigated the potential biases introduced by the researchers’ interpretations. Third, the 5-min threshold for hypotension may neglect brief but true hypotensive insults as well as artefacts. However, in the absence of concurrent detailed medical annotation, we judged it more reliable a priori to classify episodes under 5 min as artefactual, providing a conservative but robust account of hypotension in the ICU. Alternatively, key strengths include use of a mixed-methods approach to provide a comprehensive picture of current practices, and collection of blood pressure data at 1-min intervals to capture brief insults.

The optimal MAP target varied by patient, yet target personalisation remained limited in clinical practice. Target adherence also varied, with observed MAP both exceeding and failing to reach set targets. Future research will explore the feasibility and clinical implications of achieving tighter blood pressure control within a quality improvement framework.
